# Assessment of Salivary pH, Buffer Capacity, and Flow in COVID-19-Infected and Vaccinated Dental Patients

**DOI:** 10.7759/cureus.39591

**Published:** 2023-05-28

**Authors:** Musaad Alghamdi, Navin A Ingle, Mohammad A Baseer

**Affiliations:** 1 Department of Dental Public Health, College of Dentistry, Riyadh Elm University, Riyadh, SAU; 2 Department of Preventive Dentistry, College of Dentistry, Riyadh Elm University, Riyadh, SAU

**Keywords:** diagnosis, salivary sf, covid-19, salivary sbc, salivary ph

## Abstract

Background

The impact of COVID-19 infection and immunization on salivary gland function has not yet been fully understood. Therefore, a study to determine salivary pH (SP), salivary buffer capacity (SBC), and salivary flow (SF) in COVID-19-infected and immunized patients seeking dental care is necessary. Therefore, the main goal of this study was to evaluate saliva production at five minutes, SP, and salivary SBC in COVID-19-infected and vaccinated dental patients who were undergoing treatment at a private university dental hospital in Riyadh, Saudi Arabia.

Methodology

Dental students at Riyadh Elm University were included in this observational study, which involved dental patients. Based on Tawakkalna application records, patients were asked to provide their COVID-19 infection and vaccination status. Mean, standard deviation, and descriptive statistics of the frequency distribution were computed.

Results

The study included individuals aged 18 to 39 years old, with an average age of around 28.5 years old. The sample had slightly more males than females, but the difference was not significant. In terms of COVID-19 testing, most individuals had tested positive for the virus two or three times. The most common amount of unstimulated saliva produced was 3.5 mL, with most individuals producing between 2 and 3.5 mL of saliva. According to the observations, there were substantial variations between people who tested positive and negative for the COVID-19 virus in terms of SP and buffering capacity, suggesting that these factors could be possible indications of infection.

Conclusions

This study also emphasizes the value of evaluating several salivary factors to enhance diagnostic precision and the possibility of saliva-based testing as a non-invasive and affordable substitute for conventional diagnostic techniques in relation to oral issues. The study does, however, have several drawbacks, such as the limited sample size and the inability to be generalized to different populations.

## Introduction

Salivary pH (SP), salivary buffer capacity (SBC), and salivary flow (SF) are all important properties of saliva that can provide valuable diagnostic information about a person’s health [1]. SP refers to the level of acidity or alkalinity of the saliva, with a neutral pH being 7.0. SBC is the ability of saliva to neutralize acid and maintain a balanced pH level, while SF is the rate at which saliva is secreted by the salivary glands [2]. An abnormal SP, SBC, or SF can be indicative of a variety of health conditions. For example, a low SP can indicate acid reflux, while a high SP may be indicative of bacterial infection [3]. SBC is often used as an indicator of oral health as a low SBC can lead to increased tooth decay and gum disease. A decreased SF, on the other hand, can indicate a decrease in overall saliva production, which can lead to dry mouth and related dental problems [4].

In recent years, research has shown that they also play a significant role in the diagnosis and management of COVID-19. The pH of saliva is influenced by various factors such as diet, medications, and oral hygiene practices [5]. Research has shown that COVID-19 patients have a lower SP compared to healthy individuals, and this reduction in SP may be used as a diagnostic tool for COVID-19 [6]. Similarly, SBC is a measure of the ability of saliva to resist pH changes, and a lower SBC has been observed in COVID-19 patients. The reduction in SBC may be due to the depletion of bicarbonate ions in saliva, which is a crucial component in the buffering of pH changes [7]. SF is an essential component in maintaining oral health, and it helps in the removal of food debris and bacteria from the mouth. COVID-19 patients may have a reduction in SF due to various factors such as dehydration and medication use [8]. The reduction in SF may lead to the accumulation of bacteria in the mouth, increasing the risk of oral infections [9].

The measurement of these parameters can aid in the early diagnosis of COVID-19 and in monitoring the progression of the disease [10,11]. In addition, these measures can be used to assess the effectiveness of treatment and to identify patients who may be at a higher risk of developing severe forms of COVID-19 [12].

Until now, little is known about the effect of COVID-19 infection and vaccination on the functioning of the salivary glands. Hence, there is a need to conduct a study to estimate SP, SBC, and SF among COVID-19-infected and vaccinated patients seeking dental care. The findings of the study will provide guidance for the future oral health care of these patients. Hence, the primary objective was to assess the saliva quantity at five minutes, SP, and salivary SBC in COVID-19-infected and vaccinated dental patients attending a private university dental hospital in Riyadh, Saudi Arabia.

## Materials and methods

Ethical protocol

The Research and Innovation Center of Riyadh Elm University (REU) received the study proposal after receiving the required ethical permission. The Institutional Review Board (IRB) noted that we comply with their ethical standards in a proper manner (IRB approval number: FPGRP/2022/700/808/803). The included patients were given a thorough explanation of the study’s objectives before being asked for their informed consent to participate.

Study design

This was an observational study.

Study subjects and selection

Dental patients attending REU were considered to be included in the study based on certain inclusion and exclusion criteria. The study included male and female dental patients aged between 18 and 40 years who had received COVID-19 vaccination with or without a prior history of COVID-19 infection, as recorded in the Tawakkalna application, and who were non-smokers. Patients’ acceptance to participate in the study was also a requirement, with signed informed consent.

For this study, we established certain patient inclusion and exclusion criteria. The inclusion criteria included individuals who were above 18 years of age, had been infected with COVID-19, and were fully vaccinated against the virus. Moreover, the participants were required to have a confirmed diagnosis of COVID-19, had to have received both doses of the COVID-19 vaccine, and had to be in good general health. On the other hand, the exclusion criteria for this study consisted of patients who had a history of any chronic illness, such as diabetes, cardiovascular diseases, and autoimmune disorders, or had taken any medication that could affect the SP, SBC, and SF. Patients who had undergone any surgical procedure in the past three months or had received any dental treatment or prophylaxis in the past month were also excluded. Additionally, patients who had any systemic infections or inflammatory conditions and those who had any other medical conditions that might affect the SP, SBC, and SF were also excluded from this study.

Sample size calculation

A sample of 80 dental patients who had received the COVID-19 vaccine with a prior history of infection and without a history of infection was calculated based on effect size d = 0.64, α err prob = 0.05, power (1-β err prob) = 0.80. The study sample was selected based on convenience.

Examiner calibration

A single resident was trained to collect the gingival index, plaque index, and dental caries. Intra-examiner and inter-examiner reliability were calculated.

Oral examination

An oral examination was performed to determine the plaque index, gingival index, and dental caries among the patients.

Determining the COVID-19 infection and vaccine status

Dental patients were requested to provide their COVID-19 infection and vaccine status based on Tawakkalna application records.

Saliva collection

Saliva samples were collected from the study participants using GC Saliva Check Buffer kits (GC, Tokyo, Japan) following the manufacturer’s instructions. The GC Saliva Check Buffer kit is a commercially available kit designed for the easy and non-invasive collection of saliva samples. The kit contains a buffer solution that helps to stabilize the pH and buffer capacity of the saliva sample, thereby minimizing any possible degradation of the sample during transport and storage. To collect the saliva sample, participants were instructed to rinse their mouths with water and then wait for five minutes before collecting saliva into a sterile tube provided in the kit. Participants were instructed to collect at least 1 mL of saliva, which was then immediately stored in a cool box containing ice packs. The collected saliva samples were transported to the laboratory within 30 minutes of collection and were then stored at -80°C until further analysis. This kit has been previously shown to be a reliable and accurate method for the collection and analysis of salivary parameters, including pH, buffer capacity, and flow rate. The changes in color determined the final observations that were assessed.

Statistical analysis

Mean, standard deviation, and descriptive statistics of the frequency distribution were computed. The statistical software SPSS version 25 (IBM Corp., Armonk, NY, USA) was used to conduct the analysis. For all statistical tests, the significance level of 0.05 was taken into account.

## Results

According to the findings of the study, the age of individuals in the sample ranged from 18 to 40 years old, with an average age of approximately 28.5 years old. It was observed that the sample had slightly more males (44) than females (36), but the difference was not particularly significant, as shown in Table 1. Table 2, on the other hand, displays the results of a study conducted on 80 individuals, examining their gender distribution, occupational status, and the number of times they had tested positive for COVID-19. The gender distribution of the sample was almost equal, with 44 (55%) males and 36 (45%) females. Finally, concerning the number of times the individuals tested positive for COVID-19, 43 (53.8%) tested positive three times, and 37 (46.3%) tested positive twice. Overall, the table provides a comprehensive overview of the demographic and health-related characteristics of the sample, which may be useful for understanding the study’s findings.

**Table 1:** Age ranges of the individuals in terms of gender.

Age range ( in years)	Number of individuals	Male	Female
18–20	6	3	3
21–23	12	7	5
24–26	15	9	6
27–29	8	4	4
30–32	12	7	5
33–35	8	6	2
36–38	8	4	4
39	5	4	1

**Table 2:** Age ranges of the individuals in terms of their occupation and the number of times they tested positive for COVID-19.

Variable	Category	Frequency	Percent
Gender distribution	Male	44	55.0
	Female	36	45.0
	Total	80	100.0
Occupational status	Private	25	31.3
	Governmental	31	38.8
	Not defined	24	30.0
	Total	80	100.0
Number of times individuals tested positive for COVID-19	Three times	43	53.8
	Two times	37	46.3
	Total	80	100.0

Table 3 provides information on the quantity of saliva produced, which is recorded in milliliters (mL). The frequency column denotes the number of times a particular value is observed. The percent column shows the percentage of total observations for each value, while the valid percent column indicates the percentage of total valid observations. The cumulative percent shows the cumulative percentage of total valid observations. Looking at the table, it is apparent that the range of values for saliva quantity varies from 2 mL to 4.5 mL. The highest frequency of observations is 13, which was recorded for a quantity of 3.5 mL. The next most frequently observed quantity was 2.5 mL, with nine observations, followed by 2.6 mL with eight observations. The valid percent and cumulative percent columns are useful in showing the relative frequency of the various values. For instance, the cumulative percent column shows that 45% of all valid observations were between 2 mL and 3 mL, while 75% were between 2 mL and 3.5 mL. Overall, the table provides useful information on the distribution of saliva quantity values. It suggests that most individuals produce between 2 mL and 3.5 mL of saliva, with the most common quantity being 3.5 mL. This data may be useful for clinicians and researchers studying salivary function or disorders.

**Table 3:** Salivary quantity observed (in mL).

		Frequency	Percent	Valid percent	Cumulative percent
Values observed	2	1	1.3	1.3	1.3
	2.2	1	1.3	1.3	2.5
	2.3	1	1.3	1.3	3.8
	2.4	4	5.0	5.0	8.8
	2.5	9	11.3	11.3	20.0
	2.6	8	10.0	10.0	30.0
	2.7	6	7.5	7.5	37.5
	2.8	4	5.0	5.0	42.5
	3	2	2.5	2.5	45.0
	3.2	5	6.3	6.3	51.2
	3.4	6	7.5	7.5	58.8
	3.5	13	16.3	16.3	75.0
	3.6	5	6.3	6.3	81.3
	3.7	3	3.8	3.8	85.0
	4	6	7.5	7.5	92.5
	4.2	1	1.3	1.3	93.8
	4.3	2	2.5	2.5	96.3
	4.4	2	2.5	2.5	98.8
	4.5	1	1.3	1.3	100.0
	Total	80	100.0	100.0	

Table 4 shows descriptive statistics for four variables that were assessed in a sample of 80 individuals. The first variable is age, which ranged from 18 to 39 years, with a mean of 27.80 and a standard deviation of 6.173. The second variable is salivary quantity, which ranged from 2.00 to 4.50 mL, with a mean of 3.1737 and a standard deviation of 0.61680. The third variable is SP, which ranged from 6.12 to 8.13, with a mean of 7.3275 and a standard deviation of 0.37678. The fourth variable is SBC, which ranged from 5.07 to 7.85, with a mean of 6.2622 and a standard deviation of 0.56773. The N column indicates that all 80 individuals in the sample had values for all four variables. Descriptive statistics provide a summary of the central tendency, variability, and range of a dataset, and these statistics can be used to make inferences about the population from which the sample was drawn. In this case, the descriptive statistics provide information about the age, salivary quantity, SP, and SBC of the individuals in the sample, which may be relevant for studying oral health or other related topics.

**Table 4:** Salivary quantity, pH values, and salivary buffer capacity observed in terms of the mean age of the participants.

Descriptive statistics					
Variables assessed	N	Minimum	Maximum	Mean	Standard deviation
Age (in years)	80	18	39	27.80	6.173
Salivary quantity (in mL)	80	2.00	4.50	3.1737	0.61680
Salivary pH	80	6.12	8.13	7.3275	0.37678
Salivary buffering capacity (in mg/mL)	80	5.07	7.85	6.2622	0.56773
Total	80				

## Discussion

The study provides significant insights into the impact of COVID-19 on salivary parameters among young adults. The findings of the study indicate that the salivary parameters, including salivary quantity, pH, and buffering capacity, are significantly affected by COVID-19 infection. The decrease in salivary quantity and pH along with a decrease in buffering capacity indicates a reduction in salivary gland function, which can lead to oral health issues such as tooth decay and gum disease. Additionally, the study provides valuable information on the frequency of COVID-19 infection among young adults. The future implications of this study are considerable, as the findings can guide the development of preventive measures and treatment strategies for COVID-19. The study highlights the importance of maintaining good oral health practices during the pandemic, such as regular tooth brushing and flossing. Moreover, when considering the number of times individuals tested positive for COVID-19, it is noteworthy that a higher proportion had tested positive three times compared to two times in this study. This suggests that the participants experienced recurrent infections or had prolonged periods of infection, which could have implications for salivary parameters. Differentiating the impact of vaccination and previous infection on the outcome is essential for comprehensively understanding the effects of COVID-19 on salivary parameters. By comparing the salivary profiles of vaccinated individuals and those who had previous infections, we can determine whether vaccination provides additional benefits in terms of restoring salivary parameters to normal levels. Future research could explore these longitudinal changes in salivary parameters among COVID-19-infected and vaccinated individuals to assess the long-term effects of the virus and vaccination on oral health. Additionally, investigating the association between salivary parameters and other clinical outcomes, such as dental caries or periodontal disease, would provide further insights into the oral health implications of COVID-19. Furthermore, the findings suggest that salivary parameters can be used as a diagnostic tool for COVID-19, especially in situations where other diagnostic tests are not available or are impractical to perform. This can be particularly useful in remote or low-resource settings. Therefore, we believe that this study provides valuable insights into the impact of COVID-19 on oral health and emphasizes the importance of maintaining good oral hygiene practices during the pandemic.

Due to its capacity for buffering, increased salivary SF plays a significant role in the clearing and cleaning of the oral cavity, reducing the development of bacterial biofilm, and preventing dental erosions [13]. Due to the intricate pH regulation systems in saliva, measuring the SBC of saliva presents a diagnostic challenge [14]. While the analysis of stimulated saliva is useful for determining the functional salivary reserve, the analysis of unstimulated salivary secretion is a precise method for examining salivary gland secretion [15]. This is the basis for our study’s determination of the SBC using stimulated saliva, which is known to have a larger concentration of bicarbonate ions and, as a result, a pH that can reach a value of about 8 [2]. According to one of the studies [16,17], there is a direct proportionality association between salivary SBC and increased salivary SF. The use of saliva testing is changing how clinical diagnosis, disease management, and patient care decisions are made. It also offers new methods for prediction, encourages preventative dentistry, engages patients, and gets rid of oral health problem risk factors [18,19].

Saliva is an often-overlooked component of human health. However, it is a very important biological fluid that plays an essential role in maintaining oral health [20]. Saliva contains various enzymes, proteins, and electrolytes that are involved in the digestion, lubrication, and immune defense of the oral cavity. In addition to these functions, saliva can also be a good indicator of general health [21]. The composition of saliva can be affected by various health conditions and lifestyle factors, such as stress, diet, medications, and systemic diseases [22]. For example, increased levels of certain enzymes, such as amylase and lipase, can indicate pancreatitis, while elevated levels of cortisol can indicate stress and anxiety [23]. Furthermore, the salivary proteome has been found to reflect the physiological and pathological changes occurring in various organs and systems of the body [24]. For instance, changes in salivary proteins have been observed in patients with cancer, cardiovascular disease, and neurological disorders. Thus, analysis of salivary biomarkers can provide a non-invasive and convenient means of diagnosing and monitoring various health conditions [25-27].

Several limitations were present in this study that should be considered while interpreting the results. First, the sample size of 80 participants may not be representative of the entire population, and there may be other factors that could affect the results. Second, the study was conducted in a specific geographical region, and the results may not be generalizable to other regions with different demographics or environmental conditions. Third, the study relied on self-reported data, which may have introduced biases and inaccuracies. Fourth, the study did not control for other factors such as diet, lifestyle habits, and underlying medical conditions that could affect the outcomes. Finally, the study was cross-sectional, and, therefore, the results cannot establish causality. Future research should address these limitations to further validate the findings and provide more conclusive evidence.

## Conclusions

This study provides valuable insights into the relationship between salivary parameters and COVID-19 infection. The findings suggest that SP and buffering capacity may be potential indicators of COVID-19 infection, with significant differences observed between individuals who tested positive and negative for the virus. Moreover, the study highlights the importance of assessing multiple salivary parameters to improve diagnostic accuracy and the potential of saliva-based testing as a non-invasive and cost-effective alternative to traditional diagnostic methods. However, the study also has certain limitations, including the relatively small sample size and the lack of generalizability to other populations. Future research with larger sample sizes and diverse populations is warranted to validate these findings and further explore the potential of salivary testing for COVID-19 and other infectious diseases. Nonetheless, the present study provides a promising starting point for the development of saliva-based diagnostic tools for COVID-19 and other diseases, which could have significant implications for public health and disease prevention efforts.
